# Commentary: A Multilab Preregistered Replication of the Ego-Depletion Effect

**DOI:** 10.3389/fpsyg.2016.01155

**Published:** 2016-08-03

**Authors:** Junhua Dang

**Affiliations:** Department of Psychology, Lund UniversityLund, Sweden

**Keywords:** ego depletion, self-control, meta-analysis, pre-registered replication, multi-lab

The ego depletion effect has not been replicated by a recent project including 23 laboratories (*N* = 2141) in both English-speaking countries and non-English speaking countries (Hagger and Chatzisarantis, 2016). Although it provides seemingly robust evidence casting doubt on the existence of ego depletion, cautious attention should be paid to the effectiveness of the depleting task (i.e., *e*-crossing task) used in the replicating project.

The *e*-crossing task invented by Baumeister and colleagues has three main features (Baumeister et al., 1998). First, the depletion condition includes more complex rules of crossing than does the control condition. Second, participants in the depletion condition first establish a habit of crossing out every *e* and then have to override these habitual responses given more complex rules. This switching procedure is absent in the control condition in which participants cross out every *e* throughout the task. Third, the text in the depletion condition requires closer attention because of its poor legibility. However, the *e*-crossing task in Hagger et al.'s replicating project only taps the first feature and may not work as an effective depleting task. As will be shown below, this concern is indeed supported a complementary analysis of the replicating data.

To do this, datasets from different labs are collapsed into one single dataset (*N* = 2058). Note that Schlinkert, Schrama, and Koole's replication is not included because the four manipulation check items are absent in their dataset. One participant in Rentzsch, Nalis, and Schütz's replication has been excluded because the score on the fatigue item is “25,” which is beyond the range of the scale. As can be seen in Supplementary Table 1, the effect sizes are very similar to those resulting from Hagger et al.'s meta-analysis. Importantly, the depletion condition and the control condition do not differ with each other in one of the four manipulation check items (i.e., fatigue, *t* = 1.63, *p* = 0.104, *d* = 0.07). Although they do differ in the other three items, scores on only one item (i.e., effort) are above the midpoint the scale (i.e., “4”). That is to say, even in the depletion condition, the *e*-crossing task is generally considered not “depleting,” thus questioning the effectiveness of the depleting task.

Although the *e*-crossing task in Hagger et al.'s replicating project in general is not effective, for a subsample of individuals it might be “depleting.” That is to say, those who experienced depletion during the *e*-crossing task should have performed worse on the subsequent task, thus manifesting the typical ego depletion effect. Therefore, the interaction between experimental condition and each of the manipulation check items has been examined. Multiple regression analysis reveals a significant interaction between condition and effort, as shown in Supplementary Table 2. Scores on the effort item and experimental condition (control condition = 0, depletion condition = 1) are entered in the first step, and their interaction term is entered in the second step. In the full sample, the interaction is significant for both reaction time (RT) (β = 0.10, *p* = 0.001) and reaction time variability (RTV, defined as the sum of the sigma and tau variability parameters using ex-Gaussian modeling) (β = 0.08, *p* = 0.004). Simple slopes analysis is employed to test the interactional pattern (Aiken and West, 1991). As can be seen in Figure 1, in the control condition, effort predicts neither RTV (*t* = −0.71, *p* = 0.480) nor RT (*t* = −1.79, *p* = 0.074). However, in the depletion condition, the simple slope is positive and differs significantly from zero, for both RTV (*t* = 3.54, *p* < 0.001) and RT (*t* = 2.85, *p* = 0.004), indicating that the more effort participants exert during the initial depleting task, the worse they perform on the subsequent self-control task. This pattern holds in both English-speaking sample and non-English speaking sample. The interactions between experimental condition and other three manipulation check items are not significant.

**Figure 1:**
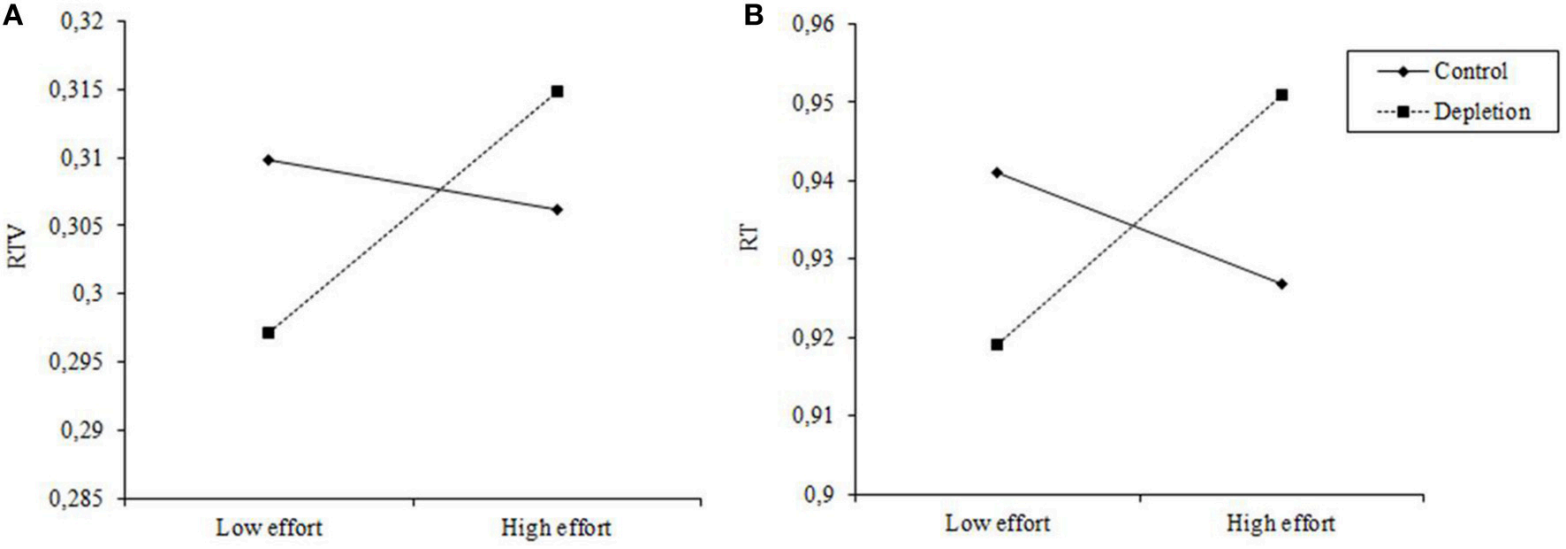
**RTV (A) and RT (B) on the subsequent task as a function of effort in the control condition and the depletion condition**.

In summary, this complementary analysis implies that the failure of Hagger et al.'s replication may result from the ineffectiveness of their manipulation. Participants generally do not consider the *e*-crossing task as “depleting.” However, for those who consider it as effortful, there is an ego depletion effect. This resonates with a recent meta-analysis of ego depletion that also points to the effectiveness of the depleting task (Dang, Unpublished data). After excluding studies using ineffective depleting tasks, a medium level of ego depletion effect was revealed by both the traditional random effects model and the newly developed method (i.e., PET-PEESE) that has been used by Carter et al. (2015). Future replications of ego depletion should take this into account and focus on the effectiveness of each depleting task frequently used in the literature. Also, the subjective ratings (i.e., effort, difficulty, fatigue, and frustration) that are commonly used might not be perfect manipulation check. Other indicators, such as attempt to overcome habits, should also be considered by future studies and replications.

## Author contributions

The author confirms being the sole contributor of this work and approved it for publication.

### Conflict of interest statement

The author declares that the research was conducted in the absence of any commercial or financial relationships that could be construed as a potential conflict of interest.

## References

[B1] Aiken L. S. West S. C. (1991). Multiple Regression: Testing and Interpreting Interactions. Newbury Park, CA: Sage.

[B2] Baumeister R. F. Bratslavsky E. Muraven M. Tice D. M. (1998). Ego depletion: is the active self a limited resource? J. Personal. Soc. Psychol. 74, 1252–1265. 10.1037/0022-3514.74.5.1252 9599441

[B3] Carter E. C. Kofler L. M. Forster D. E. McCullough M. E. (2015). A series of meta-analytic tests of the depletion effect: Self-control does not seem to rely on a limited resource. J. Exp. Psychol. Gen. 144, 796–815. 10.1037/xge0000083 26076043

[B4] Hagger M. S. Chatzisarantis N. L. D. (2016). A multi-lab pre-registered replication of the ego depletion effect. Pers. Psychol. Sci. 11, 546–573. 10.1177/1745691616652873 27474142

