# Supplementary material for: Commentary: A Multilab Preregistered Replication of the Ego-Depletion Effect
Source: Front Psychol. 2016 Aug 3;7:1155. doi: 10.3389/fpsyg.2016.01155 (PMC4971805; doi:10.3389/fpsyg.2016.01155)
Supplement: Supplementary file 1 [file Table1.DOCX]

Table 1

*Summary of the Whole Sample*

|  | Depletion | |  | Control | |  |  |  |
| --- | --- | --- | --- | --- | --- | --- | --- | --- |
| Dependent variable | *M* | *SD* |  | *M* | *SD* | *t* | *p* | *d* |
| RTV |  |  |  |  |  |  |  |  |
| Full sample | 0.31 | 0.08 |  | 0.31 | 0.08 | 0.21 | .834 | 0.01 |
| English-speaking sample | 0.32 | 0.08 |  | 0.32 | 0.08 | 0.90 | .367 | 0.06 |
| Non-English speaking sample | 0.30 | 0.07 |  | 0.30 | 0.07 | -0.41 | .683 | -0.02 |
| RT |  |  |  |  |  |  |  |  |
| Full sample | 0.94 | 0.14 |  | 0.94 | 0.14 | 0.75 | .451 | 0.03 |
| English-speaking sample | 0.96 | 0.15 |  | 0.96 | 0.14 | 0.84 | .401 | 0.06 |
| Non-English speaking sample | 0.92 | 0.13 |  | 0.92 | 0.14 | 0.29 | .775 | 0.02 |
| Effort |  |  |  |  |  |  |  |  |
| Full sample | 4.90 | 1.42 |  | 3.66 | 1.85 | 17.03 | .000 | 0.75 |
| English-speaking sample | 5.47 | 1.17 |  | 4.38 | 1.85 | 9.81 | .000 | 0.69 |
| Non-English speaking sample | 4.54 | 1.44 |  | 3.17 | 1.68 | 15.36 | .000 | 0.87 |
| Difficulty |  |  |  |  |  |  |  |  |
| Full sample | 4.00 | 1.37 |  | 1.90 | 1.03 | 39.50 | .000 | 1.75 |
| English-speaking sample | 4.08 | 1.40 |  | 1.81 | 1.03 | 26.43 | .000 | 1.86 |
| Non-English speaking sample | 3.95 | 1.35 |  | 1.96 | 1.02 | 29.45 | .000 | 1.67 |
| Fatigue |  |  |  |  |  |  |  |  |
| Full sample | 3.44 | 1.62 |  | 3.32 | 1.64 | 1.63 | .104 | 0.07 |
| English-speaking sample | 3.51 | 1.72 |  | 3.22 | 1.71 | 2.46 | .014 | 0.17 |
| Non-English speaking sample | 3.39 | 1.54 |  | 3.39 | 1.59 | -0.01 | .994 | 0.00 |
| Frustration |  |  |  |  |  |  |  |  |
| Full sample | 3.08 | 1.61 |  | 1.95 | 1.31 | 17.52 | .000 | 0.77 |
| English-speaking sample | 3.34 | 1.63 |  | 1.91 | 1.28 | 14.02 | .000 | 0.98 |
| Non-English speaking sample | 2.91 | 1.57 |  | 1.99 | 1.32 | 11.31 | .000 | 0.64 |
