# Supplementary material for: Commentary: A Multilab Preregistered Replication of the Ego-Depletion Effect
Source: Front Psychol. 2016 Aug 3;7:1155. doi: 10.3389/fpsyg.2016.01155 (PMC4971805; doi:10.3389/fpsyg.2016.01155)
Supplement: Supplementary file 2 [file Table2.DOCX]

Table 2

*The Interaction between Experiment Condition and Effort Rating*

| Variables | RTV (*β*) | RT (*β*) |
| --- | --- | --- |
| Full sample |  |  |
| Step 1 |  |  |
| Effort | .04 | .00 |
| Condition | -.01 | .01 |
| Step 2 |  |  |
| Effort × Condition | .08*** | .10*** |
| English-speaking sample |  |  |
| Step 1 |  |  |
| Effort | .02 | -.03 |
| Condition | .03 | .04 |
| Step 2 |  |  |
| Effort × Condition | .09* | .10* |
| Non-English speaking sample |  |  |
| Step 1 |  |  |
| Effort | -.02 | -.05 |
| Condition | .00 | .03 |
| Step 2 |  |  |
| Effort × Condition | .08** | .10*** |

* *p* < .10; ** *p* < .05; *** *p* < .01.
